# The National Dementia Workforce Study: Overview

**DOI:** 10.1111/jgs.70061

**Published:** 2025-09-01

**Authors:** Donovan T. Maust, James Wagner, Sheryl Zimmerman, Laura M. Wagner, Steven C. Marcus, Elizabeth M. White, Rachel S. Tocco, Joanie Rothstein, Johanna van Tyen Silbersack Hickey, Amy R. Pettit, Joanne Spetz

**Affiliations:** ^1^ Department of Psychiatry University of Michigan Ann Arbor Michigan USA; ^2^ Center for Clinical Management Research, VA Ann Arbor Healthcare System Ann Arbor Michigan USA; ^3^ Institute for Social Research University of Michigan Ann Arbor Michigan USA; ^4^ Cecil G. Sheps Center for Health Services University of North Carolina at Chapel Hill Chapel Hill North Carolina USA; ^5^ Department of Community Health Systems, School of Nursing University of California San Francisco San Francisco California USA; ^6^ Philip R. Lee Institute for Health Policy Studies University of California San Francisco San Francisco California USA; ^7^ Healthforce Center at UCSF University of California San Francisco San Francisco, California USA; ^8^ School of Social Policy and Practice University of Pennsylvania Philadelphia Pennsylvania USA; ^9^ Department of Health Services, Policy, and Practice Brown University School of Public Health Providence Rhode Island USA; ^10^ Independent Consultant Boston Massachusetts USA

**Keywords:** dementia, direct care worker, nurse, physician, workforce

## Abstract

Meeting the care needs of people with dementia is a major public health and societal challenge. In 2020, over 6 million Americans had Alzheimer's disease or a related dementia, and this is projected to increase to 14 million by 2060. Their care needs incur nearly $30,000 more in mean annual costs per person compared to peers without dementia, and overall costs are expected to rise to $1.6 trillion within 15 years. People with dementia experience poorly coordinated care, high emergency department use, elevated hospitalization rates, and substantial variation in care. Gaps in training and high workforce turnover rates, particularly in home care and residential settings, may contribute to these outcomes. Providing quality care for dementia requires a well‐trained, multidisciplinary workforce, but we lack a thorough understanding of the professionals currently providing care and the extent to which their training and composition influence outcomes. The National Dementia Workforce Study (NDWS) aims to investigate these issues on an unprecedented scale through a series of comprehensive annual surveys of dementia care providers across multiple care settings, rooted in a conceptual framework inspired by the Donabedian and Quality Health Outcomes models. Our goals are to generate a new data infrastructure and, by integrating novel survey data with other data sources, equip the research community with new tools to study relationships between workforce characteristics, care processes, and outcomes for people with dementia. Findings will provide insights into workforce dynamics, training needs, and care quality and inform strategies to improve dementia care policy, workforce capacity, and practice.

**Summary**

- Key points
  ○ A large body of research indicates that the U.S. healthcare system is not prepared to address the complex needs of people with dementia, resulting in care that is poorly coordinated, of poor quality, and high cost.
  ○ The dementia care workforce includes an array of roles, including physicians, advanced practice providers, registered and licensed practical nurses, and direct care workers (personal care aides, nursing assistants, and home health aides); the latter are the largest group of workers who provide paid care, but there has been little systematic study of their characteristics and influence on patient outcomes.
  ○ Funded by a cooperative agreement from the National Institute on Aging, the National Dementia Workforce Study (NDWS) aims to generate and make available—at no cost—the most comprehensive data to date on the U.S. dementia care workforce through implementation of annual surveys targeting key providers in various settings (community‐based care, nursing homes, assisted living, and home care) and through a large‐scale qualitative data collection effort.
- Why does this paper matter?
  ○ The NDWS will generate novel survey and interview data that are designed to be integrated with additional data sources, including Medicare claims, to enable researchers to gain critical insights into workforce dynamics, training needs, and structural inequities and into the relationship between these factors and patient outcomes.
  ○ Findings from studies using NDWS data could inform policy and practice strategies to enhance care quality for the growing population of people with dementia.

## 1 Introduction

Meeting the medical, behavioral, psychosocial, and functional needs of the nation's growing population of people with dementia is one of the top public health and societal challenges facing the U.S., and this challenge will intensify in the coming decades. In 2020, over 6 million Americans had Alzheimer's disease or a related dementia (ADRD), and that number is expected to more than double to nearly 14 million by 2060 [1]. Due to their substantial care needs, the mean cost of medical and other purchased care for a person with dementia is nearly $30,000 higher than for a similar older adult without dementia [2]. Accounting for the cost of unpaid family caregiving, overall costs totaled $915 billion in 2010 and are anticipated to rise to $1.6 trillion by 2040 [2].

The U.S. health care system is ill‐equipped to meet the complex needs of people with dementia [3], who suffer from poorly coordinated care [4], higher emergency department use [5], and higher rates of both potentially preventable and all‐cause hospitalizations [6]. Not only do people with dementia experience worse care than their counterparts without dementia overall, but the limited data available reveal enormous variation in the care received. A 2013 review of dementia care practices by primary care clinicians, for example, included just 12 studies, all of which were cross‐sectional surveys. The percentage of physicians who reported the use of diagnostic imaging ranged from 33% to 80%, while the percentage who reported making routine referrals to community services ranged from 26% to 83% [7]. Examination of cholinesterase inhibitor prescribing provides further evidence of significant variation in treatment practices by both geography (i.e., 32%–66% of people with dementia received prescriptions across hospital referral regions) and by prescriber specialty [8]. In the nursing home setting, staffing characteristics are associated with overall resident health [9, 10, 11], and facility quality may vary with the proportion of residents with dementia [12, 13]. Improving our understanding of the complex factors driving variations in care is a key part of increasing the consistency and quality of care delivery and of improving care outcomes.

### 1.1 The Dementia Workforce

Individuals with dementia receive health care and personal care in multiple settings from an especially large and diverse workforce of clinicians and care workers that includes physicians, nurse practitioners, physician assistants, and registered and licensed practical nurses [14]. In addition to these clinicians, the largest dementia workforce segment by far is “direct care” workers, including 2.4 million personal care aides, 1.1 million nursing assistants, and 800,000 home health aides [15]. Direct care workers support people with dementia across multiple settings, including nursing homes, assisted living communities, and in people's homes. Unfortunately, exceptionally high turnover is pervasive: a 2021 report examining national employee‐level Payroll Based Journal data found that nursing homes had an annual median facility turnover rate of 102.9% and 79.8% for registered and licensed nurses, respectively; 98.8% for certified nursing assistants; and 128% for total nursing staff [16]. While maintaining an adequate and stable workforce is challenging across all settings, it is likely to be a particular challenge for home‐based care: in keeping with findings that many aging adults have a strong preference to remain in their own home [17], the U.S. Bureau of Labor Statistics projects growth of 36% for personal care aides and even more for home health aides in the next decade [15].

The dementia workforce also faces issues related to variable readiness to diagnose and care for people with dementia. Whereas some professional roles have extensive education and practical experience as a prerequisite, others do not and may include minimal formal or on‐job training. Across roles, findings suggest that much of the workforce providing care to people with dementia perceives gaps in their training and lacks confidence in providing dementia care. In a national survey of primary care clinicians, for example, only 21% reported feeling highly confident in their ability to correctly recognize when a patient has cognitive impairment; most referred individuals to specialists, whose numbers are limited. When asked about confidence educating patients and their families about dementia management and care, only 25% of primary care clinicians felt extremely confident [18]. In qualitative interviews with 39 PCPs about their role in providing care to people with dementia, the majority identified gaps and elaborated on specific needs to improve care, including the need to feel more confident in their ability to make a diagnosis without specialist input and to address system constraints such as limited appointment time, reimbursement, and staff support [19].

Finally, the professional dementia care workforce is under stress. For example, dementia care workers may experience physical behaviors (e.g., hitting, biting) or defensive actions (e.g., shoving, rejection of help) from people with dementia with agitation or confusion, including incidents that cause injury [20, 21]. Workers from minoritized racial and ethnic backgrounds and immigrant populations—who are over‐represented in the nursing home workforce, where many people with dementia receive care [22]—may need to navigate a lack of cultural and language concordance between themselves and the people for whom they provide care [23]. Unfortunately, many nursing staff report discrimination and racism from residents with dementia [24]. This potentially challenging environment may contribute to documented widespread turnover (i.e., replacement) among nursing home workers [16] and means that organizations face a constant challenge to find new staff who are either well‐prepared or can be trained to meet residents' care needs. At the same time, little is known about factors that contribute to positive work environments for dementia care workers, such as adequacy of wages, sick leave, and career growth opportunities. Further, whereas workforce stress and turnover have been most frequently studied in the nursing home setting, similar factors are at play in assisted living and home care settings. Improving stability of staffing is integral to high‐quality care, as staffing levels, turnover, and absenteeism all influence outcomes for people with dementia [12, 25].

### 1.2 Addressing Barriers to Workforce Research

Whereas a vast body of literature has focused on family and other unpaid caregivers who meet critical needs for people with dementia, we know far less about the professional workforce and the extent to which their training and composition influence outcomes. This research has been difficult to accomplish for several reasons. First, the size and complexity of the workforce and its distribution across multiple settings make it difficult to find, sample, and reach workers in order to generate nationally representative estimates. Second, both generating and analyzing data for this workforce require a deep understanding of multiple issues, including clinical features of dementia, factors affecting clinical care delivery and practice patterns, training requirements across professions, organizational functioning, market conditions, health care economics, and advanced sampling and analytic methods.

To overcome these barriers, the National Institute on Aging (NIA) has entered into a large, 5‐year cooperative agreement with a unique, multidisciplinary, national team of experts to: (1) conceptualize and launch the National Dementia Workforce Study (NDWS); (2) develop an associated data infrastructure and library of resources to facilitate the use of the resulting data by the broad research community; and (3) support and expand the community of investigators who are focused on investigating dementia workforce issues and their relationship to the quality of dementia care. This initiative brings together clinicians, health services researchers, quantitative and qualitative methodologists, health economists, and scholars with expertise in related fields (medical anthropology, sociology, demography, biostatistics, implementation science, bioethics)—along with input from people with dementia, their caregivers, and the organizations that serve them—from across the United States. The NDWS was funded in September 2023 and has launched the first national surveys focused specifically on the professional dementia care workforce in the U.S.: the Community Clinician Survey, Nursing Home Staff Survey, Assisted Living Staff Survey, and Home Care Staff Survey, with five waves of each planned through 2028. As part of a *Journal of the American Geriatrics Society* special collection, we provide an overview of these efforts here.

## 2 Methods

### 2.1 Conceptual Model

Our approach to understanding the dementia care workforce is rooted in the classic Donabedian model of health care, which conceptualizes care as consisting of structures and processes that result in patient outcomes [26]. Structures are the context in which care is delivered, including but not limited to the physical setting, equipment, financial resources, and staff ratios and training. Processes are technical and interpersonal health care actions (e.g., testing, physical exams, assessments), including actions related to diagnosis, treatment, and patient education. Patient outcomes include various aspects of health status, ranging from an acute infection or exacerbation of a chronic condition to experiencing an injurious fall.

**FIGURE 1:**
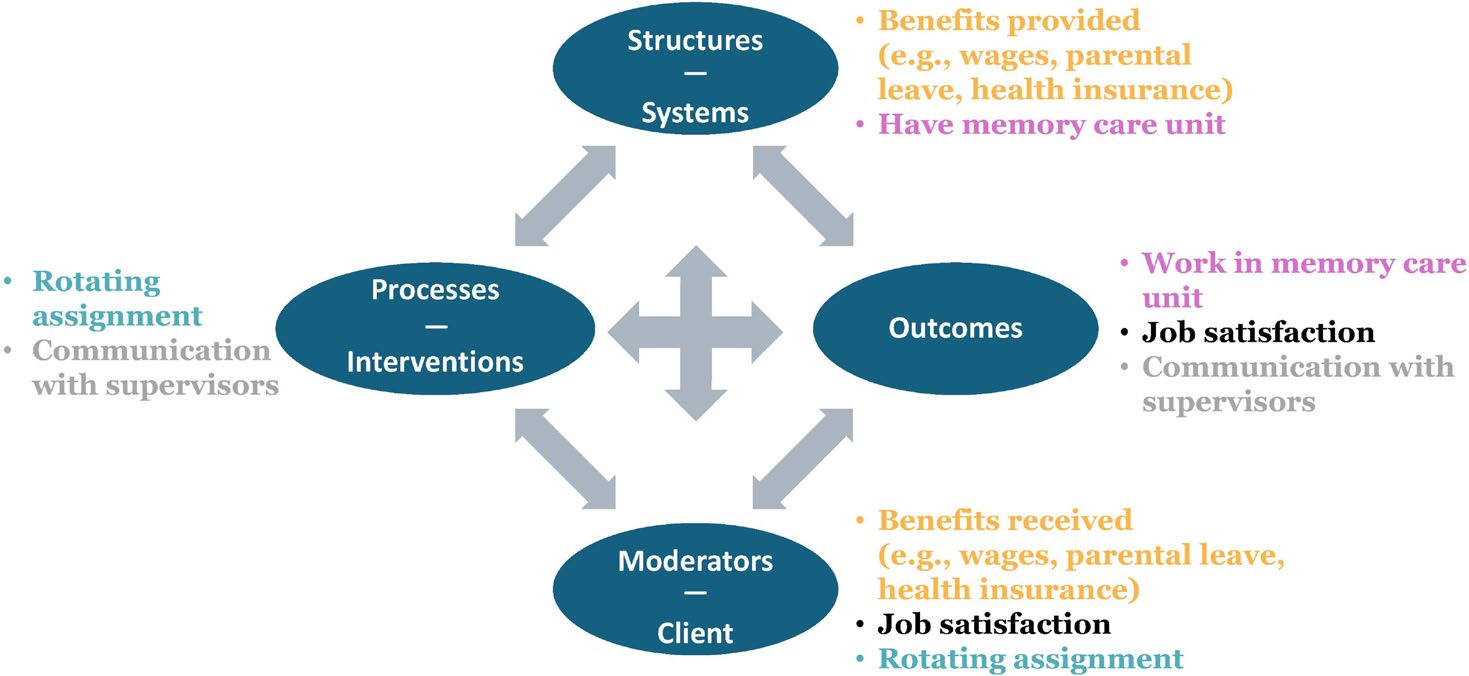
Donabedian and Quality Health Outcomes Model as adapted for the National Dementia Workforce Study. Specific concepts are shown as belonging to more than one domain, as their conceptualization may vary depending on a researcher's interests. For example, while job satisfaction may be conceptualized as an outcome of interest in one study, in another study it may be a factor that moderates the impact of a particular care process on a different outcome of interest (e.g., communication with supervisors).

Because the Donabedian model views structures, processes, and outcomes as a linear pathway (structures ➔ processes ➔ outcomes) and does not account for the fact that each of the three domains may have direct effects on—or function as moderators of—the others, we have also incorporated the Quality Health Outcomes (QHO) model [27]. The QHO model accounts for similar domains as Donabedian (i.e., structure/system, process/interventions, outcomes) but allows for reciprocal interaction and expressly recognizes the operative role of moderators, or characteristics of those to whom an intervention is targeted (i.e., “client” domain in the QHO model). Through application of the QHO model, a patient outcome (e.g., experiencing a fall) may impact care processes (e.g., patient education) and also organizational structures (e.g., hiring patient educators), especially if the patient caseload is one of individuals at high risk of falls (i.e., a moderator). The model provides flexibility to view certain key concepts (e.g., job satisfaction) as an outcome in some analyses or as a moderator that may influence care recipient outcomes in others. Figure 1 illustrates five items from the NDWS Staff surveys that could potentially be assigned to multiple domains in the QHO model, depending on researchers' questions of interest. Ultimately, however, we anticipate that each researcher using NDWS data will select the appropriate conceptual model for their analysis based on their specific topic, disciplinary expertise, and methods.

### 2.2 Core NDWS Components

#### 2.2.1 Overview

The four NDWS surveys (Community Clinician, Nursing Home Staff, Assisted Living Staff, and Home Care Staff) will be conducted annually for 5 years. Given the critically important issue of turnover among the professional dementia care workforce, the surveys are also designed to be longitudinal in order to track entries into and exits from the professional dementia workforce in each of these settings. This offers the capacity to generate crucial insights into modifiable factors that drive workforce turnover and guide future efforts to reduce turnover.

Survey data will be available to researchers in a de‐identified, publicly available form through the NIA‐supported National Archive of Computerized Data on Aging (NACDA), while unmodified data and all linked data sources will live within the secure NIA‐supported Linked Information for Knowledge on Aging Enclave (LINKAGE) platform. On LINKAGE, researchers will be able to link survey data with a variety of other data sources, including person‐level Medicare and Medicaid claims and Minimum Data Set (MDS) assessments, which researchers can use to objectively examine care delivered to people with dementia; additional details are provided below. Both the de‐identified and linked data will be available at no cost.

In addition to primary survey data collection, the NDWS team will also complete semi‐structured interviews with a large subset of respondents from each of the four NDWS surveys and with administrators of organizations that are part of the sample frames (planned *N* = 1000 interviews total). These qualitative data will also be available to the research community.

Finally, NDWS includes a Research Studies Core that plans to fund up to six pilot awards per cycle, of approximately $100,000 each in direct costs. This component seeks to advance the NIA Alzheimer's Disease/Alzheimer's Disease and Related Dementias (AD/ADRD) Research Implementation Milestones and to accelerate the use of NDWS data.

**FIGURE 2:**
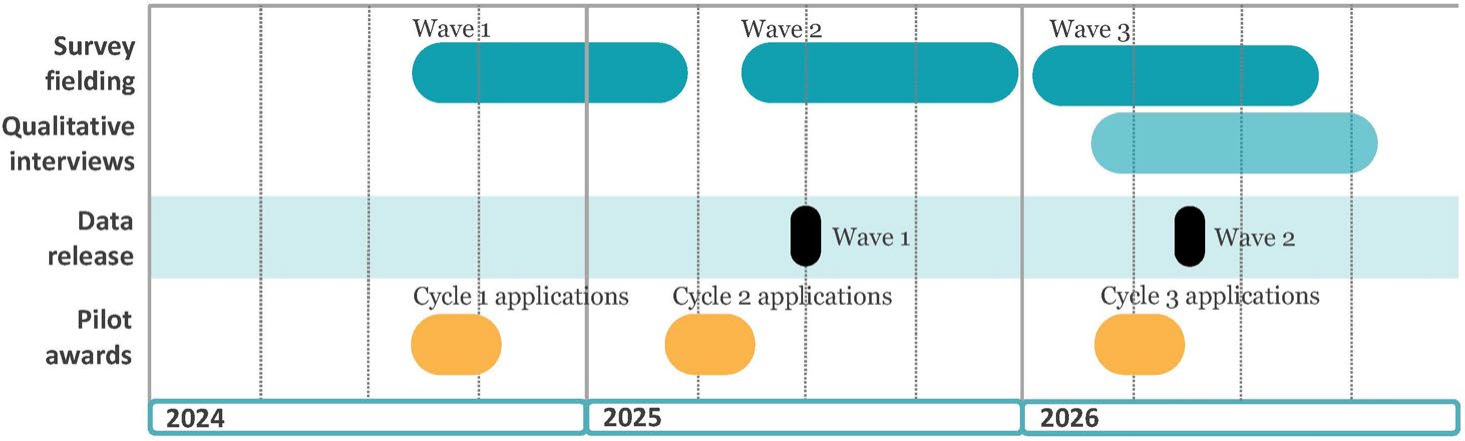
Timeline of NDWS survey fielding, data release, and pilot awards. The figure presents a high‐level overview of the project timeline (dotted vertical lines within each calendar year reflect quarters), The timeline was compressed in the first 2 project years given the necessary start‐up activities. The data release for each Wave will include updates of the linked files outlined in Table 1. The general timing presented in 2026 will be repeated in 2027 and 2028 for Waves 4 and 5 of data collection, release, and pilot award applications.

Extensive project information, including survey instruments and guidance on data access, is available on the NDWS website (www.ndws.org). A high‐level timeline of project activities is presented in Figure 2.

#### 2.2.2 Study Settings, Populations, and Sampling Approaches

The Community Clinician Survey was designed to include clinicians who provide “dementia care”—that is, those who would potentially screen for and diagnose cognitive impairment and then help people with dementia and any care partners address cognitive and functional decline. The sample frame, derived from provider data drawn from national Medicare and Minimum Data Set (MDS) claims and assessment data, includes primary care physicians and nurse practitioners (including those specialized in geriatrics); psychiatrists and psychiatric‐mental health nurse practitioners; neurologists; and non‐surgical physician assistants. The Community Clinician Survey includes clinicians who also provide care in residential and/or inpatient settings (i.e., it is not limited to those with only office‐based practice). Whereas people with dementia see clinicians from a variety of other specialties (e.g., cardiologists, nurse anesthetists), these clinicians were not included in the sample because it is unlikely that dementia is a primary focus of the care they provide. Data collection may expand to include them in the future. In the first wave, 25,000 community clinicians were invited to participate.

As noted, the other NDWS surveys focus on nursing home, assisted living, and home care settings and staff. People with dementia comprise a large proportion—if not the majority—of residents in most nursing homes [13], and 44% of assisted living residents are estimated to have dementia [28]. We focus on the largest occupations within these key practice settings: licensed nurses (registered nurses and licensed practical/vocational nurses) and the direct care workforce (nursing assistants, personal care aides, and others). Sample frames were constructed to be nationally representative of the components of the dementia care workforce in each setting (e.g., representing all licensed nurses working in assisted living).

Sampling for the Nursing Home Staff, Assisted Living Staff, and Home Care Staff Surveys was designed to begin at the organizational level, with randomly sampled staff within each employing organization invited to participate. In the initial years of NDWS, we focus on licensed nurses and direct care workers in each of these three settings. By the end of the 5‐year project, the planned total survey samples will be drawn from a total of 700 organizations (e.g., nursing homes, assisted living communities, or home health and home care agencies) for each survey: Nursing Home Staff (*N* = 19,140), Assisted Living Staff (*N* = 15,660), and Home Care Staff (*N* = 20,560). After the first wave, each subsequent round of annual staff surveys will include a mix of new respondents and longitudinal follow‐up in order to track workforce entrances and exits. Finally, for each organization that participates in the staff survey, we will field an administrator survey at intake that captures additional information about organizational practice setting and characteristics. (The administrator survey will not be longitudinal).

Additional information about the sampling, design, and fielding of all surveys is available in companion articles in this special collection [29, 30, 31].

#### 2.2.3 Study Domains

Each NDWS survey is designed to generate insights into training, compensation, payment models, and care practices of each of the respective workforce groups. Two other articles in this special collection describe the process for developing the Community Clinician survey instrument and the Nursing Home, Assisted Living, and Home Care questionnaires, respectively [29, 30].

#### 2.2.4 Qualitative Data

Beginning in Year 3, we will expand our primary data collection beyond the surveys to include semi‐structured interviews with a subset of survey respondents from each wave. We will make the resulting qualitative data publicly available along with the survey data. By the end of the project, we plan to complete approximately 1000 interviews, leading to one of the largest bodies of qualitative data available for research in the U.S. To facilitate researchers' use of this invaluable data source, we will apply analytic tools, such as machine learning and natural language processing, to help researchers identify interviews that address particular domains of interest. These qualitative data can then be leveraged by researchers to yield additional insights not found in the structured survey data, and their inclusion in NDWS is designed to attract qualitative and mixed‐methods researchers in a way that is unique among other NIA‐funded or workforce surveys.

#### 2.2.5 Planned Data Linkages

The NDWS surveys and qualitative data are designed to be useful both as standalone data sources and to be linked to other major datasets, to enable objective assessment of the relationships between workforce issues and clinical outcomes. In addition, data linkages will enable examination of important location‐specific factors, including health care supply, socioeconomic resources, and the policy environment that may influence both the workforce and the care it is able to deliver. Table 1 summarizes data linkages that we expect to be available based on the location of the survey respondent (e.g., Area Health Resources Files for respondent county) or their Centers for Medicare and Medicaid Services (CMS) identifier.

**TABLE 1:** Data available to link with NDWS surveys.

Data sources	Survey				Sample content
	Community Clinician	Nursing Home Staff	Assisted Living Staff	Home Care Staff	
Local context					
Area Health Resources Files (county‐level)	**✓**	**✓**	**✓**	**✓**	Health care professions, health facilities, hospital use and expenditures
Social Vulnerability Index (county‐level)	**✓**	**✓**	**✓**	**✓**	Composite measure of disadvantage developed by the CDC and based on U.S. Census data (e.g., percentage living in poverty)
AARP Long‐Term Services and Supports Scorecard (state‐level)	**✓**	**✓**	**✓**	**✓**	Indicators of: affordability and access, choice of setting and provider, safety and quality, support for family caregivers, and community integration
Medicaid Long‐Term Services and Supports Annual Expenditures Report (state‐level)	**✓**	**✓**	**✓**	**✓**	Medicaid spending, including on institutional services and home and community‐based services
CMS data					
Medicare, Medicaid, and MDS data (beneficiary‐level)	**✓**	**✓**	Planned^a^	**✓** ^b^	Diagnoses, services, and other specific claims data
Physician and Other Practitioner files (NPI‐level)	**✓**	n/a	n/a	n/a	Generated from the Carrier file, summarizes all services billed to Medicare by NPI
Medicare Part D Prescribers (NPI‐level)	**✓**	n/a	n/a	n/a	Generated from Part D, summarizes all prescription claims prescribed by NPI
Nursing Home Compare (CCN‐level)	n/a	**✓**	n/a	n/a	Number of certified beds, quality measure scores, staffing levels
Payroll Based Journal files (CCN‐level)	n/a	**✓**	n/a	n/a	Hours paid for each staff reporting category (e.g., Registered Nursing, Certified Nurse Aides)
Care Compare: Home Health Quality Reporting (CCN‐level)	n/a	n/a	n/a	**✓** ^b^	Quality measures at the agency level
Other					
LTCFocus (CCN‐level)	n/a	**✓**	n/a	n/a	Care quality, resident composition
LTC Data Cooperative (CCN‐level)	n/a	Partial^c^	Partial		Record data for participating nursing home and assisted living organizations
NDWS‐prepared claims‐ and assessment‐based summary files (NPI‐ and CCN‐level)	**✓**	**✓**	Planned^a^	Planned^b^	Panel characteristics and services summarized at the NDWS‐participating respondent and organization levels

Abbreviations: CCN: CMS Certification Number; CDC, Centers for Disease Control and Prevention; CMS, Centers for Medicare and Medicaid Services; LTC, long‐term care; MDS, Minimum Data Set; NPI, National Provider Identifier.

^a^ Will be available for people with dementia in larger assisted living communities with a unique 9‐digit zip code; resident beneficiaries can be identified by their residence zip code in the CMS enrollment file.

^b^ Available where the participating organization is CMS‐certified to provide home health services.

^c^ Available where NDWS respondents are employees of nursing homes and assisted living facilities participating in the LTC Data Cooperative, and the latter has approved the research project.

Since many researchers may not have the capacity or expertise to work with raw claims or assessment data, NDWS will use CMS claims and assessment data to prepare annual summary files capturing key demographics, aspects of care, and outcomes among people with dementia who are cared for by NDWS survey respondents. Initially, these will be available for the Community Clinician Survey (e.g., number of home visits to people with dementia; racial composition of people with dementia within clinician panels; number of people with dementia with a claim for an advance care planning visit) and for the Nursing Home Staff Survey (e.g., mean age and racial composition of residents with dementia; number of residents with self‐ or staff‐reported pain). The availability of these summary files will enable research teams to more readily explore a variety of research questions, such as whether patients of clinicians in rural settings are less likely to receive neuroimaging or whether residents of nursing homes that offer dementia training programs are prescribed fewer psychotropic medications.

In future years, NDWS will expand the linked data available so that additional data sources of high interest are available to researchers.

## 3 Discussion

During the past several decades, the NIA has made significant investments in data collection and data infrastructure to support researchers' investigations into aging and dementia. Insights from these individual sources of data—including nationally representative surveys such as the Health and Retirement Study and the National Health and Aging Trends Study—can be further expanded by linking them with additional data sources (e.g., Medicare claims and Census data) to generate fundamental new knowledge related to aging and health. The National Dementia Workforce Study builds on these efforts and, for the first time, reflects a significant investment into understanding the workforce that is providing care to older Americans, specifically those with dementia.

As the population of Americans with Alzheimer's disease or a related dementia continues to grow, ensuring that there are sufficient professionals to care for them is an urgent public health need. This came into sharp focus during the COVID‐19 pandemic [32], when strain on the health care system led to significant burnout, retirement, and attrition in the health care workforce as a whole, and in settings providing care to older adults (such as nursing homes) in particular. A comprehensive understanding of the workforce providing care to people with dementia—including the heterogeneous array of providers, who number in the millions and include physicians, nurse practitioners, physician assistants, registered nurses, licensed practical nurses, nursing assistants, personal care aides, and home health aides—is an essential first step in ensuring both adequacy and quality of care. This knowledge, along with a more thorough understanding of both barriers to optimal workplace conditions and training and facilitators of positive work environments and performance, is a prerequisite for efforts to expand the workforce to meet demand and to increase evidence‐based care and decrease potentially harmful practices. This is especially true given that major implementation science frameworks highlight collaboration with the individuals delivering care as a critical element of any practice change efforts [33].

Critically, the NDWS infrastructure will enable survey respondent data to be linked to claims or assessment data of people with dementia (e.g., Medicare, Medicaid) to observe objective measures of the care delivered. This will afford the remarkable opportunity to examine how workforce characteristics are associated with actual care delivery (e.g., lecanemab prescribing, hospice enrollment) and outcomes (e.g., avoidable hospitalizations, nursing home placement) experienced by people with dementia. NDWS will also offer researchers a unique opportunity to access a large repository of qualitative interview data from survey respondents and administrators from sampled organizations, enabling qualitative and mixed‐methods studies that have not been possible with other NIA‐funded or workforce surveys. In addition to these qualitative data being another rich source for researchers, the interviews will also allow the study team to obtain participant feedback on topics such as recruitment and questionnaire content that can inform NDWS going forward.

Despite the unprecedented scope of this initiative, we note some limitations. People with dementia receive care from an enormous variety of clinicians and in an array of settings [14], and practical and financial limitations during project launch required some boundaries around who we could reach in our initial efforts. In particular, adult day centers, Programs of All‐Inclusive Care for the Elderly (PACE), hospitals, and hospice are all important settings of care, but the sample frame is not designed to capture clinicians who work exclusively in these settings at this time. Similarly, individuals with dementia may have multiple clinical comorbidities that require important subspecialist care, but the Community Clinician Survey currently focuses on primary care (including geriatrics), psychiatry, and neurology. The role of other specialties (e.g., cardiologists, intensivists) is an area for future consideration. We also acknowledge that other professionals are integral to optimal care delivery to people with dementia, such as social workers, psychologists, physical therapists, and occupational therapists. Finally, NDWS does not include primary data collection from people with dementia or their caregivers, which was explicitly not requested by NIA as part of this cooperative agreement. The future direction of NDWS, including the clinicians and settings that may be captured in later years, are part of ongoing conversations between the project team, staff at the NIA, and the research community. Our initial efforts will lay the groundwork for this future inquiry.

As the largest initiative of its kind to date, the National Dementia Workforce Study represents a significant investment by the NIA. At the end of the 5‐year award period, we will have launched a family of four surveys aiming for over 70,000 contacts collected from over 40,000 unique respondents, along with rich qualitative data from a subset of respondents—all of which will be available to researchers, at no cost, with a host of rich linked data sources. This data infrastructure will be accompanied by myriad efforts to facilitate and promote its use, allowing researchers to use this unprecedented resource to conduct a wide range of important studies seeking to understand the reciprocal links between the dementia care workforce and the adults to whom they provide care, generating data‐based approaches to better support these clinicians and the individuals and families they serve.

## Author Contributions

Concept and design: All authors. Acquisition, analysis, or interpretation of data: n/a. Drafting of the manuscript or revising critically for important intellectual contributions: All authors. Final approval of the published version: All authors.

## Conflicts of Interest

The authors declare no conflicts of interest.

## Linked Articles

This publication is part of the special collection of National Dementia Workforce Study. To view all articles under this special collection visit https://agsjournals.onlinelibrary.wiley.com/doi/toc/10.1111/(ISSN)1532‐5415.national‐workforce‐study.
